# Identification and functional characterization of lactic acid bacteria with probiotic potential for alleviating premenstrual syndrome symptoms

**DOI:** 10.3389/fmicb.2026.1866966

**Published:** 2026-07-09

**Authors:** So-hyun Yoon, Lee Ching Lew, Yong-Ha Park, Irfan A. Rather

**Affiliations:** 1 School of Biotechnology, Yeungnam University, Gyeongsan, Gyeongbuk, Republic of Korea; 2 Probionic Corp., Jeonbuk Institute for Food-Bioindustry, Jeonju-si, Jeollabuk-do, Republic of Korea; 3 Department of Biological Sciences, Faculty of Science, King Abdulaziz University, Jeddah, Saudi Arabia; 4 Centre of Excellence in Bionanoscience Research, King Abdulaziz University, Jeddah, Saudi Arabia

**Keywords:** health, lactic acid bacteria, premenstrual syndrome, probiotics, vaginal microbiota

## Abstract

**Introduction:**

Premenstrual syndrome (PMS) is a relatively widespread disorder associated with cyclical hormonal oscillations and disruptions in microbial homeostasis, particularly involving β-glucuronidase –producing bacteria that facilitate estrogen reabsorption. This study aimed to isolate, identify and functionally characterize probiotic strains from the vaginal microbiota of healthy Korean women in order to evaluate their potential in mitigating PMS-associated pathophysiology.

**Materials and methods:**

A total of 12 isolates, named as KVS strains, were evaluated for their probiotic potential, including antimicrobial, enzyme inhibitory, and anti-adhesive activities associated with PMS. These isolates were further evaluated for adhesion capacity, survival under acidic conditions, auto-aggregation ability, inhibition of β-glucuronidase activity, antimicrobial activity, and suppression of Gardnerella vaginalis adhesion to HeLa cells.

**Results and discussion:**

Several KVS strains, such as KVS001, KVS002, KVS004, KVS006, and KVS008 showed strong probiotic potential, adhesion capacity, high auto-aggregation rates, and survival in acidic conditions. In addition, these strains showed high antimicrobial activity and significantly suppressed β-glucuronidase activity, suggesting a mechanism for reducing estrogen recirculation. Moreover, many isolates effectively inhibited G. vaginalis adhesion to HeLa cells, indicating strong potential to counteract dysbiosis-associated PMS triggers. Collectively, the findings identify multiple KVS strains as promising probiotic candidates with multifunctional mechanisms relevant to PMS alleviation, supporting their prospective development as health-functional foods or therapeutic agents targeting microbiome-mediated hormonal modulation.

## Introduction

Reproductive health is a state of complete physical, mental, and social wellbeing regarding reproductive structure and function. It is not merely the absence of disease or dysfunction but a holistic concept that underpins the health and wellbeing of individuals, families, and societies (Lee and Ju, 2015; Lee et al., 2021; Cha and Jung, 2023). Recognized as a cornerstone of social and economic development, reproductive health directly influences lifelong health outcomes, quality of life, and overall societal progress (Lee and Ju, 2015; Lee et al., 2021). Globally, the significance of reproductive health has been emphasized through initiatives such as the United Nations’ Millennium Development Goals, which highlight gender equality and the empowerment of women, alongside national policies and research initiatives aimed at improving women’s health (Lee and Ju, 2015). In Korea, governmental programs largely focus on fertility stabilization and childbirth support, yet there remains a relative lack of attention to common reproductive disorders and their symptom management. Among these, premenstrual syndrome (PMS) and bacterial vaginosis (BV) are two prevalent conditions that substantially affect women’s daily functioning, mental health, and quality of life, underscoring the urgent need for effective, safe, and sustainable interventions.

Premenstrual syndrome is a cyclic disorder characterized by recurring physical, emotional, and behavioral symptoms, typically arising during the luteal phase of the menstrual cycle. While many women experience mild symptoms, a significant proportion suffer severe discomfort that interferes with personal, social, and occupational activities (Bertazzoni Minelli et al., 1996; Girman et al., 2003; Choi et al., 2010; Noviyanti et al., 2021; Yadav, 2025). The etiology of PMS is multifactorial, involving psychological factors such as stress and personality traits, as well as physiological imbalances, particularly in sex hormones such as estrogen and progesterone (Bertazzoni Minelli et al., 1996; Girman et al., 2003; Noviyanti et al., 2021). Recent reports suggest that the gut microbiota could possibly contribute to PMS pathophysiology through its effects on estrogen metabolism and host endocrine regulation (Ervin et al., 2019; Sui et al., 2021). Therefore, alteration in microbial composition have been reported in women with PMS, while β-glucuronidase activity due to microbiome may influence estrogen recirculation and hormonal balance (Takeda et al., 2022; Nabeh, 2023; Yao et al., 2024). Current therapeutic approaches include lifestyle modifications, stress management, and pharmacological interventions such as selective serotonin reuptake inhibitors, diuretics, and hormone-based treatments (Bäckström et al., 2003). However, these interventions often carry side effects and may not provide adequate relief for all patients, highlighting the need for safer, more holistic strategies.

On the other hand, bacterial vaginosis among women of reproductive age, arises from an imbalance in the vaginal microbiome, particularly a reduction in protective Lactobacillus species and overgrowth of anaerobic bacteria such as *Gardnerella* (Ya et al., 2010; Amabebe and Anumba, 2018; Vazquez et al., 2019). While antibiotics such as metronidazole and clindamycin are effective in treating BV, recurrence rates remain high, with studies reporting up to 30% within 3 months and 80% within 9 months (Sobel et al., 2006; Verstraelen and Verhelst, 2009; Bungau et al., 2021; Wu et al., 2022). Recurrence is often attributed to the failure of beneficial bacteria to reestablish dominance, persistent dysbiosis, and the potential development of antibiotic resistance. These limitations underscore the need for safe, long-term interventions that restore and maintain a healthy vaginal microbiome.

Probiotics are live microorganisms that confer health benefits when administered in adequate amounts, have emerged as promising candidates for managing reproductive health disorders (Sánchez et al., 2017; Krishnamoorthy et al., 2018). Probiotic strains capable of surviving gastric acidity, adhering to mucosal surfaces, and exerting antimicrobial effects can modulate both gut and vaginal microbiota, thereby influencing systemic and local physiological processes. Recent studies and meta-analyses suggest that probiotics can alleviate PMS symptoms by stabilizing intestinal microbial balance, regulating hormone levels, and reducing inflammation, while in BV, they can promote Lactobacillus colonization, lower vaginal pH, and inhibit pathogen overgrowth, potentially reducing recurrence (Campagne and Campagne, 2007; Gilbert, 2013; Chee et al., 2020). Despite these promising findings, commercially available probiotic products targeting PMS or BV remain limited, and none effectively address both conditions simultaneously. Therefore, there is a critical need to identify and screen novel probiotic strains with dual functionality, which can be developed into safe, effective health-functional foods, ultimately improving the quality of life and reproductive health of women.

In the present study, vaginal lactic acid bacteria were isolated from healthy Korean women and evaluated for probiotic properties as relevant to PMS and bacterial vaginosis. This study aimed to identify strains with strong probiotic characteristics, including epithelial adhesion, antimicrobial activity, inhibition of β-glucuronidase, hydrogen peroxide production, and antagonistic effects against *Gardnerella vaginalis*. By functionally characterizing these isolates, we sought to identify probiotic candidates with potential to support vaginal microbial balance and contribute to microbiome-mediated strategies for alleviating PMS-associated symptoms.

## Materials and methods

### Isolation and identification of KVS strains

This study was conducted with 12 strains of lactic acid bacteria isolated from six Korean women who voluntarily participated after signing a written consent form, and were named as KVS (Korean female Vaginal Secretion) strains. The participants were healthy Korean Women of reproductive age (24–34 years) and were recruited voluntarily for sample collection. None of the participants reported any clinical symptoms of vaginal infection or reproductive tract discomfort at the time of sampling, and none had received antibiotic treatment or probiotic supplementation within the preceding 3 months. Healthy participants were selected to obtain bacterial isolates representative of a stable vaginal microbiota and to minimize potential confounding factors associated with vaginal dysbiosis.

Briefly, vaginal samples were collected using sterilized cotton swabs and cultured in de-Man Rogosa, Sharpe (MRS) broth at 37 °C for 18 h. After incubation, the cultured broth was inoculated on Bromocresol Purple (BCP) agar plate and cultured at 37 °C for 18 h. After incubation, two colonies were selected from the yellow acid producing zones on the BCP agar plates based on visible colony morphology and were further sub-cultured in MRS medium under same conditions.

The isolated strains were identified by 16S rRNA gene sequencing using universal rRNA gene primers (27F, 1492R primer). Sequencing was performed by the commercial sequencing service provider Solgent. The resulting sequences were identified by comparing with the GeneBank database using the NCBI nucleotide BLAST tool. The 12 KVS strains were suspended in 30% glycerol liquid for preservation and stored at −45 °C and thawed at room temperature before use.

### Culture conditions and preparation of cell-free supernatant

To enhance the bioactivity of long-term preserved strains and select healthy strains, the culture was sub-cultured 2–3 times at 37 °C for 18 h using MRS broth (MB cell, United States). To prepare cell-free supernatant (CFS), the overnight culture was inoculated into the MRS broth 1% (v/v) and cultured at 37 °C for 18 h. Then after incubation, the centrifugation was carried out (10,000 g) at 4 °C for 5 min. The supernatant was collected and filter sterilized using 0.22 μm sterile syringe filter.

### Evaluation of probiotic properties

#### Antibiotic susceptibility test

For antibiotic susceptibility test, as suggested by the European Food Safety Authority (EFSA), antibiotic susceptibility test was done using broth microdilution method. As per EFSA guidelines, a total of nine antibiotics were tested: streptomycin, ampicillin, erythromycin, chloramphenicol, clindamycin, tetracycline, kanamycin, vancomycin, and gentamycin. The antibiotics were dissolved in MRS broth with concentrations of 2–256 μg/mL. KVS strains (5 × 10^5^ CFU) were cultured using MRS broth at 37 °C for 18 h. The culture was diluted until 10^6^ CFU/mL was achieved with 2X MRS. Each well of the microplate contained 100 μL of antibiotics and 100 μL of diluted KVS strains. Followed by incubation for 24 h at 37 °C, the MIC was determined as the lowest concentration able to inhibit the growth of the bacteria to be tested. The test was repeated three times and evaluated according to the criteria of EFSA (EFSA Panel on Additives and Products or Substances used in Animal Feed (FEEDAP), 2012).

#### Adhesion assay

To exert beneficial effects in the host, probiotics must exhibit the capacity to adhere to human intestinal and other epithelial cells. Therefore, cell adhesion assay was performed by auto-aggregation and mucin adhesion assays. Where, the former directly measures the intestinal cell adhesion, and later measures the ability of probiotics to attach to the cell with a mucus layer. In brief, auto-aggregation was evaluated as described by Al Kassaa et al. (2014). The overnight cultures of KVS strains were adjusted to an OD_600_ of 1.0 and inoculated at 1% in 5 mL MRS broth, followed by incubation for 18 h. The cultures were subjected to centrifugation (4,000 g) at 4 °C for 5 min. The pellets were washed twice using phosphate-buffered saline (PBS). The cell suspension was kept at room temperature for auto-aggregation observation. Briefly, 0.1 mL of the upper layer of suspension was mixed with 0.9 mL PBS after 5 h (A1) and immediately (A0). The auto-aggregation ability was calculated according to the following formula:


$$Autoaggregation\left(\%\right)=\left(1-\frac{A\,1}{A\,0}\right)\cdot 100$$

Similarly, mucin adhesion assay was performed as described elsewhere (Valeriano et al., 2014). A 100 μL of mucin solution (1 mg/mL) in 10 mmol/L HEPES-Hank (HH) buffer was immobilized on each well of the microplate for 1 h and incubated overnight at 4 °C. The next day, wells were washed twice with 200 μL of HH buffer and kept with 100 μL of 20 mg/mL BSA for 2 h at 4 °C. After 2 h, wells were washed twice with 200 μL of HH buffer to remove unbound BSA. Then 100 μL of each strain (10^8^ CFU) were added to wells, and cultured at 37 °C for 1 h. After incubation, wells were washed five times with 200 μL sterile citrate buffer to remove unattached bacteria. The surface adherent bacteria were released by washing the samples with 200 μL of 0.5% Triton X-100 (Valeriano et al., 2014). Th resulting bacterial suspensions were cultured on an MRS agar plate after dilution.

#### Acid and bile tolerance

To measure the acid and bile resistance of KVS strains, both assays were performed following the procedure described by Seo et al. (2012) and Ahmad Rather et al. (2013). All the strains were cultured for 18 h and subsequently centrifuged at 4,000 × g and 4 °C for 5 min. The resulting pellets were washed twice with PBS and adjusted to a concentration of 10^9^ CFU/mL. For acid tolerance test, 1 mL of standardized cell suspension was inoculated into 9 mL MRS broth acidified to pH 3.0. Similarly, for bile tolerance assay, 1 mL of standardized cell suspension was added to 9 ml MRS broth containing 0.3% bile salts. Therefore, the resulting initial assay concentration was approximately 10^8^ CFU/mL. Total viable cell counts were the determined after 3 and 6 h of incubation at 37 °C using the pour plate method.

#### Antimicrobial activity of KVS strains against PMS related pathogens

To evaluate whether the isolated KVS strains could suppress microorganism associated with microbial imbalance relevant to premenstrual syndrome (PMS), antimicrobial activity was assessed against selected pathogenic bacteria. This assay was performed to examine the potential inhibitory effects of KVS on PMS associated pathogens. Therefore, the antimicrobial activity of KVS strains was evaluated using the broth microdilution method against six pathogens associated with PMS. These pathogens primarily include; but not limited to, *Escherichia coli*, *Klebsiella pneumoniae*, *Staphylococcus aureus*, *Salmonella enterica*, *Acinetobacter baumannii*, and *Clostridium perfringens*. Antimicrobial activity was assessed by adding 100 μL of fresh culture of pathogens (5 × 10^6^ CFU/mL) and 100 μL CFS of KVS strains to each well of the microplate, followed by incubation at 37 °C for 18 h. After incubation, viable pathogen counts were enumerated on the respective media as shown in Table 1.

**TABLE 1:** Culture media and antibiotics used for the tested pathogens.

Pathogens	Culture media	Antibiotics
*Escherichia coli*	Tryptic Soy Broth	Clindamycin (32 μg/mL)
*Klebsiella pneumoniae*	Tryptic Soy Broth	Ciprofloxacin (0.4 μg/mL)
*Staphylococcus aureus*	Tryptic Soy Broth	Ciprofloxacin (0.3 μg/mL)
*Salmonella enterica*	Tryptic Soy Broth	Kanamycin (0.3 μg/mL)
*Acinetobacter baumannii*	MacConkey	Ciprofloxacin (4 μg/mL)
*Clostridium perfringens*	Reinforced clostridial medium (DIFCO)	Ciprofloxacin (5 μg/mL)

#### Enzyme inhibition assay

Since β-glucuronidase contributes to estrogen deconjugation and reabsorption, inhibitory activity against this enzyme was evaluated to examine a potential microbiome-mediated mechanism relevant to PMS. This assay was performed to determine whether the KVS strains exhibited inhibitory activity against β-glucuronidase, an enzyme implicated in estrogen deconjugation and reactivation and therefore associated with the pathophysiology of PMS (Sui et al., 2021). The assay employed the culture supernatant of *E. coli*, which is known to produce the enzyme extracellularly (Martins Perin et al., 2010).

For sample preparation 500 μL of *E. coli* (5 × 10^5^ CFU), 250 μL CFS of KVS strains or control (10 mM L-aspartic acid or MRS), and 0.1 M phosphate buffer were mixed in a 1.5 mL microcentrifuge tube and incubated at 37 °C for 18 h. Following incubation, the cultures were centrifuged (10,000 × g) at 4 °C for 5 min, and the resulting supernatants were collected and stored at −45 °C.

To quantify β-glucuronidase inhibition, 235 μL of the collected supernatant and 15 μL of PNPG (*p*-nitrophenyl glucuronide, 3.15 mg/mL) were added to microplate wells and incubated at 37 °C for 1 h. Absorbance was measured at 405 nm. The inhibition rate was calculated as the following formula:


$$Inhibitionrate\left(\%\right)=\left\{\frac{\left(c\,o\,n\,t\,r\,o\,l\,O\,D-s\,a\,m\,p\,l\,e\,O\,D\right)}{c\,o\,n\,t\,r\,o\,l\,O\,D}\right\}\cdot 100$$

#### Antimicrobial activity against bacterial vaginosis associated pathogens

To investigate whether the isolated KVS strains could contribute to maintenance of vaginal microbial balance, antimicrobial activity was evaluated against *Gardnerella vaginalis* KCTC 5096, one of the predominant pathogens associated with bacterial vaginosis. Therefore, all KVS strains were tested against this pathogen. Briefly, 100 μL of pathogen suspension (10^5^ CFU/ml) and 100 μL of CFS of KVS strains were added to each well of a microplate and cultured at 37 °C for 18 h. Following incubation, the viable cell counts of *G. vaginalis* were determined by plating on Brucella agar.

#### Assessment of hydrogen peroxide production by KVS strains

Hydrogen peroxide production was evaluated because it is an important functional characteristic of vaginal lactic acid bacteria and contributes to suppression of pathogenic microorganisms in the vaginal environment. The reagent solution was prepared according to the method of Mančušková et al. (2016), as shown in Table 2. Solution 1 was prepared by dissolving 1 mg of *o*-dianisidine in 0.4% Triton X-100. (final concentration 3.2 mM). Solution 2 was prepared by dissolving 120 μg of peroxidase in 10 mM phosphate buffer (final concentration 120 μg/mL or 24 U/mL).

**TABLE 2:** Composition of the reagent solutions used for hydrogen peroxide measurement.

Component	Volume
Solution 1	1 mL
Solution 2	0.3 mL
0.4% Triton X-100	0.2 mL
10 mM phosphate buffer	18.5 mL

The hydrogen peroxide production by the KVS strains was measured following the method described by Mančušková et al. (2016). The strains were cultured in MRS broth at 37 °C for 18 h. After incubation, the cultures were centrifuged (2,000 × g) at 4 °C for 10 min, and the supernatant was collected. Subsequently, 0.2 mL of the supernatant was mixed with 0.8 mL of the reagent solution and incubated at 25 °C for 15 min. After incubation, the absorbance was measured at 430 nm.

#### Inhibition of *Gardnerella vaginalis* adhesion to HeLa cells

Adhesion of *G. vaginalis* to epithelial cells is an important early event in the development of bacterial vaginosis. Therefore, one of the main approaches in the treatment of BV is the reduction of the proportion of *G. vaginalis* in the vagina. This test was performed to measure the inhibition ability of KVS strains on the adhesion of *G. vaginalis* to HeLa cells.

The HeLa cell line was obtained from the Korean Cell Line Bank (Seoul National University, Korea). The cells were derived from cervical cancer cells and cultured in RPMI 1640 containing 25 mM HEPES, 300 mg/mL L-glutamine, 10% FBS, 25 mM NaHCO_3_, and 1% antibiotics (final concentration: 100 U/mL penicillin and 100 μg/mL streptomycin) at 37 °C under 5% CO_2_.

The inhibition assay for *G. vaginalis* adhesion was performed according to the method of Choi et al. (2022). HeLa cells were seeded into 6-well cell culture plates at density of 5 × 10^4^ cells/mL and incubated at 37 °C under 5% CO_2_ conditions for 2 days. The adherent HeLa cells were washed twice with sterile PBS. After washing, the KVS strain suspension (1 mL, 1 × 10^9^ CFU/mL) together with *G. vaginalis* suspension (1 ml, 5 × 10^5^ CFU/mL) were added to each well. The plates were incubated at 37 °C under 10% CO_2_ for 1 h. After incubation, the HeLa cells were washed three times with sterile PBS and detached from the microplate using cell scrapers. The recovered cell suspension was plated onto Brucella agar plate containing 5% sheep blood and 1% ampicillin and incubated for 2 days. After incubation, the number of viable cell count of adherent *G. vaginalis* was then determined.

On the other hand, adhesion to epithelial cells is an important probiotic characteristic associated with colonization and persistence within the vaginal mucosal environment. Therefore, the adhesion ability of KVS strains to HeLa cells was evaluated.

The adhesion to the HeLa cell line was assayed according to the method of Petrova et al. (2016). Cultured HeLa cells were washed twice with pre-warmed 1X PBS. Then, 3 × 10^7^ CFU/mL bacterial suspension was inoculated into each well and cultured at 37 °C for 1 h. After 1 h, the cells were washed again with pre-warmed PBS to remove non adherent bacteria. Subsequently, 100 μL of 0.25% trypsin–EDTA was added to each well, and the plates were incubated at 37 °C for 10 min in a humidified atmosphere containing 5% CO_2_. Finally, 900 μL of PBS was added, and the suspension was homogenized. Serial dilutions were then prepared and plated onto MRS agar to quantify the viable bacterial cell counts adhering to the HeLa cells.

### Statistical analysis

All experiments were performed in triplicate, and the results are presented as mean ± standard deviation (SD). Statistical analyses were conducted using GraphPad Prism version 10.0. Differences among groups were evaluated by one-way analysis of variance (ANOVA) followed by Tukey’s multiple comparison test. Values of *p* < 0.05 were considered statistically significant.

## Results

### Isolation and identification of strains

A total of 12 strains were isolated from vaginal samples obtained from 6 healthy Korean women who participated voluntarily. These isolates were designated as KVS (Korean Vaginal Secretions). Healthy women were selected to obtain bacterial isolates representative of a non-dysbiotic vaginal microbiota and to facilitate identification of probiotic candidates associated with vaginal health.

The result of molecular characterization by 16S rRNA gene sequencing are shown in Table 3. Of the 12 isolates, 8 strains were identified as *Lactobacillus* species (KVS001, KVS004, KVS006, KVS008, KVS009, KVS010, KVS011), while KVS002 was identified as *L. rhamnosus* and deposited as *L. rhamnosus* probio-99 to Korean Collection of Type Cultures under the accession number KCTC15528BP. In addition, three strains were identified as *Enterococcus* species (KVS005, KVS007, and KVS012). One strain, KVS003, was identified as *Acinetobacter johnsonii*, an opportunistic pathogen (Wong et al., 2017).

**TABLE 3:** Sequencing results of strains isolated from the vagina.

Samples			Species
Subject	Colony number	Sample name	
1	1	KVS001	*Lacticaseibacillus rhamnosus*
	2	KVS002	*Lacticaseibacillus rhamnosus*
2	1	KVS003	*Acinetobacter johnsonii*
	2	KVS004	*Lactobacillus reuteri*
3	1	KVS005	*Enterococcus faecalis*
	2	KVS006	*Limosilactobacillus fermentum*
4	1	KVS007	*Enterococcus faecalis*
	2	KVS008	*Limosilactobacillus fermentum*
5	1	KVS009	*Limosilactobacillus fermentum*
	2	KVS010	*Limosilactobacillus fermentum*
6	1	KVS011	*Limosilactobacillus fermentum*
	2	KVS012	*Enterococcus faecalis*

The predominance of *Lactobacillus* species among these isolates is consistent with the microbial composition typically observed in healthy vaginal microbiota. In contrast, the detection of *A. johnsonii* highlights that occasional presence of opportunistic organisms may be present even in participants without clinical symptoms of vaginal infection.

### Probiotic properties evaluations

#### Antibiotic susceptibility test

Assessment of antibiotic susceptibility was performed as part of the safety evaluation of the isolated KVS strains. Because probiotic candidates intended for human use should not serve as reservoirs of transferable antibiotic resistance genes, their susceptibility profiles were examined according to EFSA guidelines. Horizontal transfer of antibiotic resistance determinants may contribute to the emergence and dissemination of multidrug-resistant bacteria (McInnes et al., 2020). Therefore, antibiotic susceptibility represents a critical criterion in the safe commercialization of probiotic strains. According to EFSA guidelines, a strain is considered susceptible when its growth is inhibited at or below the defined cut-off concentration.

In this study, the KVS strains exhibited low resistance to ampicillin, kanamycin, streptomycin, erythromycin, clindamycin, tetracycline, and chloramphenicol, but showed high resistance to gentamycin and vancomycin (Table 4). The intrinsic resistance to vancomycin and gentamicin, which is characteristic of many Lactobacillus species, does not pose a transferable resistance risk. All 8 tested strains, KVS001, KVS002, KVS004, KVS006, KVS008, KVS009, KVS010, and KVS011, met the EFSA criteria for non-antibiotic-resistant strains and were considered suitable for potential probiotic evaluation.

**TABLE 4:** Antibiotic susceptibility of KVS strains.

KVS strains																								
	KVS001		KVS002		KVS003		KVS004		KVS005		KVS006		KVS007		KVS008		KVS009		KVS010		KVS011		KVS012	
ANTIBIOTICS	*EFSA*	*L. rhamnosus*	*EFSA*	*L. rhamnosus*	*EFSA*	*A. johnsonii*	*EFSA*	*L. reuteri*	*EFSA*	*E. faecalis*	*EFSA*	*L. fermentum*	*EFSA*	*E. faecalis*	*EFSA*	*L. fermentum*	*EFSA*	*L. fermentum*	*EFSA*	*L. fermentum*	*EFSA*	*L. fermentum*	*EFSA*	*E. faecalis*
Amp*	4	1	4	1	n.r.	n.r.	2	1	4	1	2	1	4	1	2	1	2	1	2	1	2	1	4	1
Van*	n.r.	n.r.	n.r.	n.r.	n.r.	n.r.	n.r.	n.r.	4	–	n.r.	n.r.	4	–	n.r.	n.r.	n.r.	n.r.	n.r.	n.r.	n.r.	n.r.	4	–
Gen*	16	16	16	16	n.r.	n.r.	8	2	32	64	16	16	32	64	16	16	16	16	16	16	16	16	32	64
Kan*	64	64	64	64	n.r.	n.r.	64	64	n.r.	n.r.	32	32	n.r.	n.r.	32	32	32	32	32	32	32	32	n.r.	n.r.
Str*	32	8	32	8	n.r.	n.r.	64	8	n.r.	n.r.	64	8	n.r.	n.r.	64	8	64	8	64	8	64	8	n.r.	n.r.
Ery*	1	1	1	1	n.r.	n.r.	1	1	4	1	1	1	4	1	1	1	1	1	1	1	1	1	4	1
Clin*	1	1	1	1	n.r.	n.r.	1	1	n.r.	n.r.	1	1	n.r.	n.r.	1	1	1	1	1	1	1	1	n.r.	n.r.
Tet*	8	1	8	1	n.r.	n.r.	16	2	4	2	8	2	4	2	8	2	8	2	8	2	8	2	4	1
Chl*	4	1	4	1	n.r.	n.r.	4	4	32	2	4	1	32	1	4	1	4	1	4	1	4	1	32	1

*Amp, ampicillin; Van, vancomycin; Gen, gentamycin; Kan, kanamycin; Str, streptomycin; Ery, erythromycin; Clin, clindamycin; Tet, tetracycline; Chl, chloramphenicol.

### Adhesion assay

#### Auto-aggregation and mucin adhesion assay

Adhesion related characteristics are important attributes of probiotic microorganisms, as they facilitate colonization, persistence, and competitive exclusion of pathogenic microorganism within mucosal environments. Therefore, auto-aggregation and mucin adhesion assays were performed to evaluate the mucosal adherence potential of the KVS strains. Adhesion to intestinal epithelial cells is a key determinant of probiotic strains ability to colonize the gastrointestinal tract (Kos et al., 2003). Such adhesion may prevent microbial elimination and provide a competitive adherence to other microorganisms.

**FIGURE 1:**
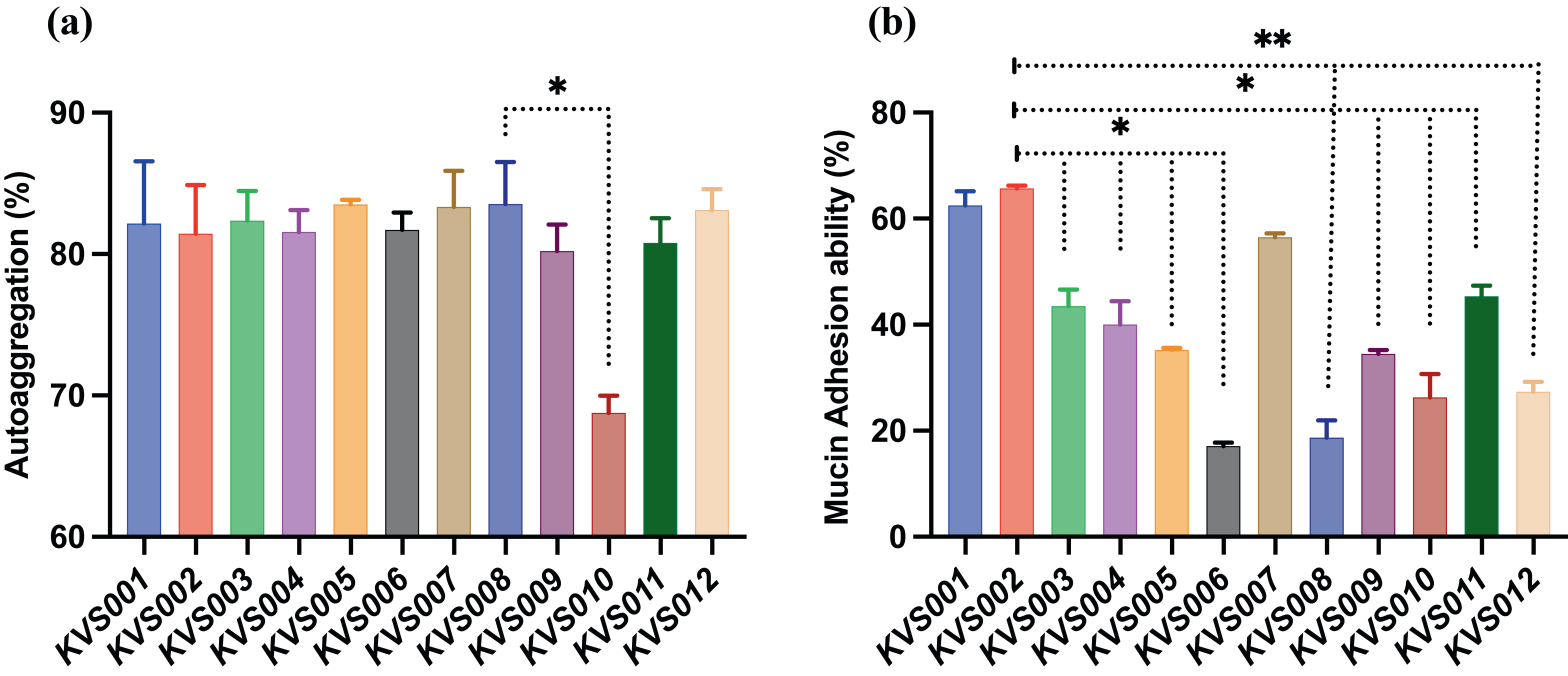
Adhesion ability of KVS strains. **(a)** Percentage of auto-aggregation. **(b)** Mucin adhesion of KVS strains (*p < 0.05; **p < 0.01; bars without any sign depicts not significant).

In this study, auto-aggregation was performed to evaluate the ability of the strains to form multicellular clumps, which indirectly reflects their potential to adhere to mucosal surfaces such as the intestinal epithelium. Figure 1a shows the results of auto-aggregation assay. The 12 strains exhibited a high level of auto-aggregation, with a mean value of 81.03%. KVS008 showed the highest aggregation capacity (83.54%), followed by KVS005 and KVS007, as 83.5 and 83.33%, respectively (*p* < 0.05). These high aggregation values indicate strong cell–cell interactions, a characteristic commonly associated with improved persistence within mucosal environment. Strains exhibiting robust auto-aggregation generally considered promising probiotic candidates because of their colonization potential.

The mucin adhesion assay was conducted to measure the adhesion ability of the strains to adhere to mucosal epithelial surfaces. High mucin adhesion suggests a greater potential for colonization of mucus-rich environments such as vaginal mucosa. The mucin adhesion capacity of the KVS strains is shown in Figure 1b. Among the 12 strains tested, KVS002 exhibited the highest mucin adhesion rate (65.67%), followed by KVS001 (62.5%) and KVS007 (59%) (*p* < 0.05). These results indicate that several KVS strains possess strong mucin-binding properties, which may support stable retention within mucosal niches. Strains with greater mucin affinity are generally more competitive in establishing persistent colonization and resisting displacement by other microorganisms.

#### Acid and bile tolerance

**FIGURE 2:**
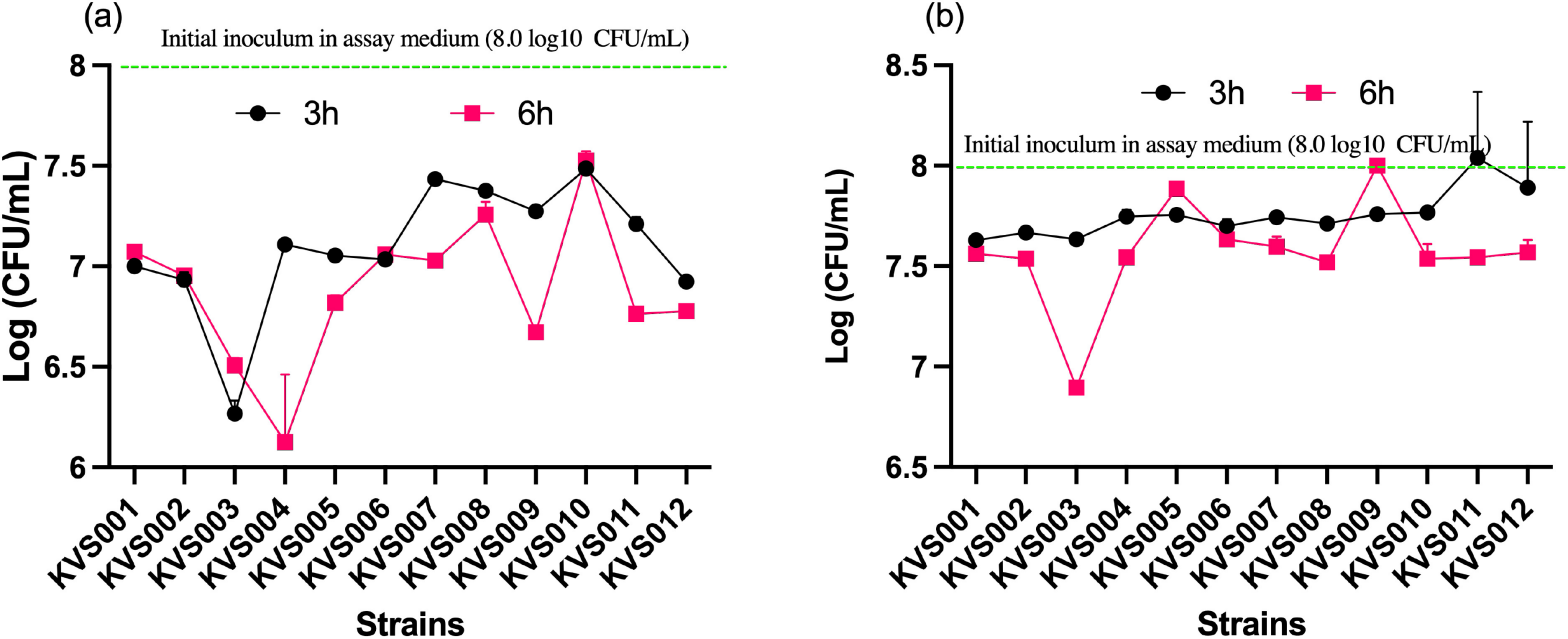
Tolerance of KVS strains after 3 h, and 6 h exposure to pH 3.0 and 0.3% bile. **(a)** Acid tolerance. **(b)** Bile tolerance. The green dotted reference line represents the initial inoculum concentration in assay medium (8.0 log_10_ CFU/mL before exposure to acid or bile.

Ingested lactic acid bacteria reach the intestines through the stomach and duodenum, where they are exposed to digestive enzymes, acid pH, and bile salts. These conditions represent major physiological stressors that can affect bacterial survival during gastrointestinal transit (Succi et al., 2005). Therefore, probiotic strains must be able to withstand these harsh condition and remain viable in order to exert their beneficial effects. Although the KVS strains were isolated from vaginal microbiota, assessment of acid and bile tolerance was performed to determine their resilience under physiological stress conditions and their potential for oral probiotic administration. While survival rates varied among strains, all KVS strains demonstrated the ability to survive under simulated gastric and intestinal conditions. The detailed survival rates under acidic and bile conditions are shown in Figures 2a,b.

The acid tolerance ability of KVS strains was evaluated using MRS adjusted to pH 3.0, as shown in Figure 2a. After 3 h of exposure, most strains exhibited low viability, with survival rates ranging from 1.85 to 30.65%. After 6 h of exposure to acidic MRS, some of the KVS strains showed much lower survivability as compared with results after 3 h; as shown by the strains KVS004, KVS005, KVS007, KVS008, KVS009, KVS011, and KVS012. Among the tested isolates, KVS010 exhibited the highest acid tolerance, retaining 30.65% viability after exposure to MRS adjusted to pH 3.0.

Notably, all 12 KVS strains exhibited strong tolerance to bile salt, as shown in Figure 2b. After 3 h exposure to MRS containing 0.3% bile salts, the viability of most strains ranged from 12.75 to 63.50%. After 6 h, 11 strains except KVS003 showed higher survival rate than at 3 h, with viability between 60 and 100%. Therefore, among the 12 strains tested, KVS009 showed the highest bile tolerance, with increased viability after 6 h, indicating a robust ability to withstand the bile salt stress.

#### Antimicrobial activity assay associated with premenstrual syndrome

**FIGURE 3:**
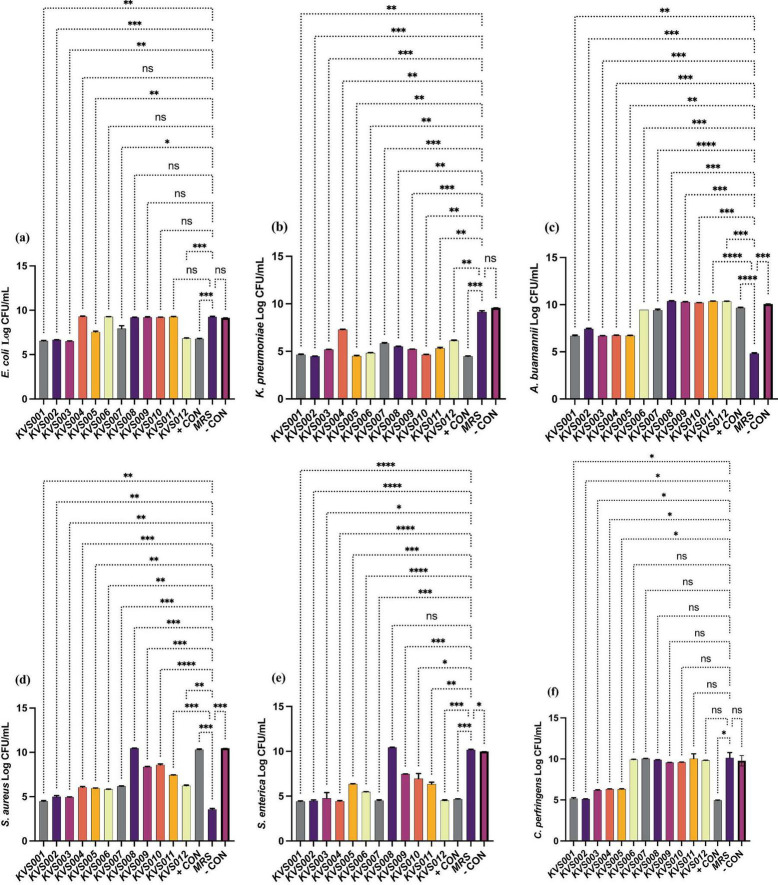
Antimicrobial activity of cell-free supernatant (CFS) produced by KVS strains against β-glucuronidase production pathogens (*p < 0.05; **p < 0.01; ***p < 0.001; ****p < 0.0001; *ns* = not significant). **(a)** ++ CON (clindamycin 32 μg/mL), MRS (MRS+ *E. coli*), -CON (PBS only). **(b)** + CON (ciprofloxacin 0.4 μg/mL), MRS (MRS + *K. pneumoniae*), - CON (PBS only). **(c)** + CON (ciprofloxacin 4 μg/mL), MRS (MRS + *A. baumannii*), - CON (PBS only). **(d)** + CON (ciprofloxacin 0.3 μg/mL), MRS (MRS + *S. aureus*), -CON (PBS only). **(e)** + CON (kanamycin 0.3 μg/mL), MRS (MRS + *S. entrica*), -CON (PBS only). **(f)** + CON (ciprofloxacin 5 μg/mL), MRS (MRS + *C. perfringens*), - CON (PBS only).

Since microbial imbalance may contribute to PMS associated symptoms, the antimicrobial activity of the KVS strains was evaluated against six pathogenic bacteria. This analysis was conducted to assess potential probiotic activity and their ability to support microbial homeostasis. The antimicrobial activity of the KVS strains against six pathogens—*Escherichia coli*, *Klebsiella pneumoniae*, *Acinetobacter baumannii*, *Staphylococcus aureus*, *Salmonella enterica*, and *Clostridium perfringens* is shown in Figures 3a–f. In case of *E. coli*, the positive control (clindamycin, 32 μg/mL) showed the strongest inhibition (*p* < 0.05), while six strains (KVS001, KVS002, KVS003, KVS005, KVS007, and KVS012) significantly reduced bacterial growth compared with the negative control. KVS003 demonstrated the highest activity (99.76%), followed closely by KVS001 (99.75%) and KVS002 (99.67%). For *K. pneumoniae*, ciprofloxacin (0.4 μg/mL) served as the positive control, and all KVS strains inhibited more than 99% of growth, with KVS001, KVS002, and KVS005 each showing 99.99% inhibition. In the case of *A. baumannii*, ciprofloxacin (4 μg/mL) produced the highest activity (*p* < 0.05), while five strains (KVS001, KVS002, KVS003, KVS004, and KVS005) significantly inhibited growth, led by KVS003 (99.89%), KVS001 (99.89%), and KVS005 (99.89%). Against *S. aureus*, ciprofloxacin (0.3 μg/mL) again showed the strongest inhibition, and 11 strains—excluding KVS008—suppressed growth by more than 99%, with KVS001, KVS003, and KVS002 each exhibiting 99.99% inhibition. For *S. enterica*, the positive control (kanamycin, 0.03 μg/mL) yielded the highest activity, and 11 strains except KVS008 inhibited more than 99% of growth, with KVS001, KVS004, and KVS012 each reaching 99.99% inhibition. Finally, against *C. perfringens*, ciprofloxacin (5 μg/mL) exhibited the greatest activity (*p* < 0.05), while five strains—KVS001, KVS002, KVS003, KVS004, and KVS005—significantly inhibited growth compared with the negative control. KVS002 showed the highest inhibition (99.99%), followed by KVS001 and KVS003 (99.99% each).

Overall, several KVS strains showed strong antimicrobial activity against the target pathogens, with inhibition rates exceeding 99% in most of the cases. Therefore, these results suggest that selected KVS strains possess antagonistic properties that may contribute to suppression of potentially harmful microorganism that are potentially associated with premenstrual syndrome (PMS).

#### Enzyme inhibition assay

β-glucuronidase is involved in the deconjugation of estrogen metabolites, thereby influencing estrogen recirculation within the body. Increased β-glucuronidase activity has been suggested to contribute to hormonal imbalances associated with PMS. Therefore, inhibition of this enzyme may represent potential mechanism for alleviating PMS related symptoms.

**FIGURE 4:**
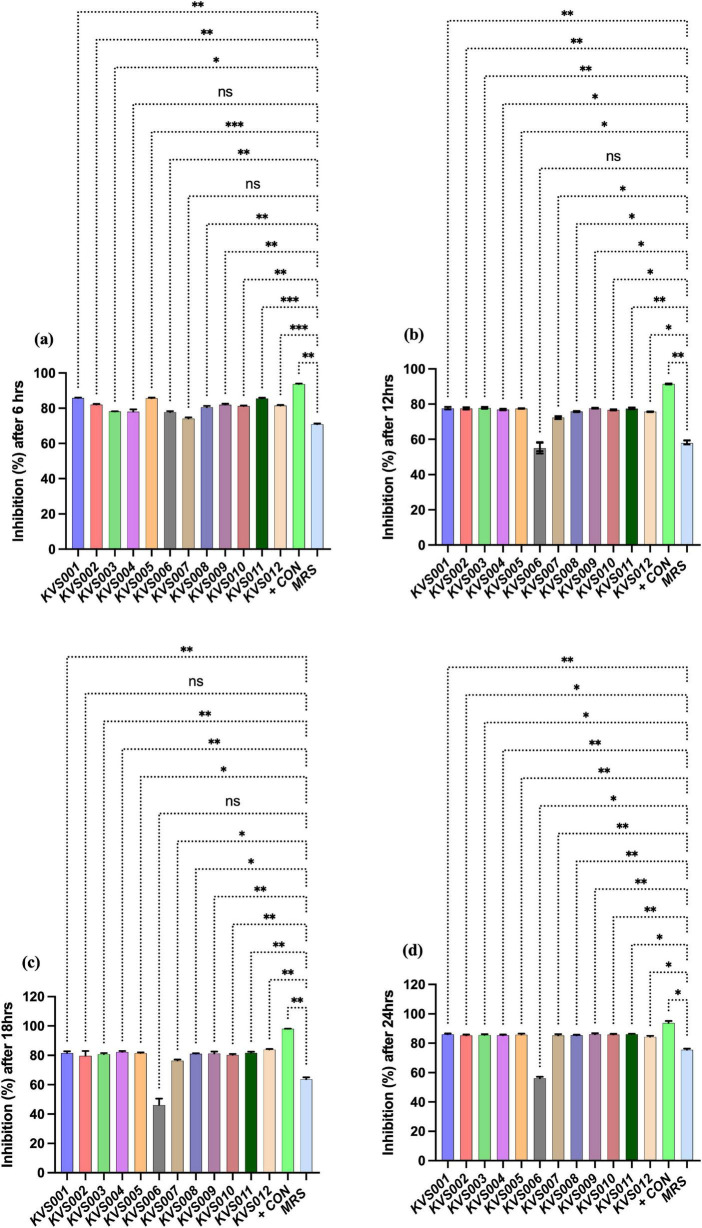
Inhibition activity of KVS strains on β-glucuronidase**. (a)** After 6 h of incubation. **(b)** After 12 h of incubation. **(c)** After 18 h of incubation. **(d)** After 24 h of incubation (*p < 0.05; **p < 0.01; ***p < 0.001; *ns* = not significant). Inhibitor 10 mM L-Asp was used as positive control (+CON). Uninoculated MRS was used as medium control.

The results of the enzyme inhibition assay are shown in Figure 4. The positive control (inhibitor, 10 mM L-Asp) showed the highest enzyme inhibition activity reducing β-glucuronidase by 99% compared to the negative control (*p* < 0.05). The MRS (blank control) showed inhibition rate of 63.67%, whereas all KVS strains, except KVS006, showed significantly higher inhibition activity (*p* < 0.05). Among the KVS strains, KVS012 showed the highest inhibition rate reducing β-glucuronidase 83.87% followed by strain KVS004 (82.20%) and strain KVS011 (81.63%) (*p* < 0.05).

These results further depict that several KVS stains possess the ability to inhibit β-glucuronidase activity and may potentially contribute to modulate the estrogen metabolism, possibly a mechanism that could be relevant to PMS management.

#### Inhibition of bacterial vaginosis associated pathogens

**FIGURE 5:**
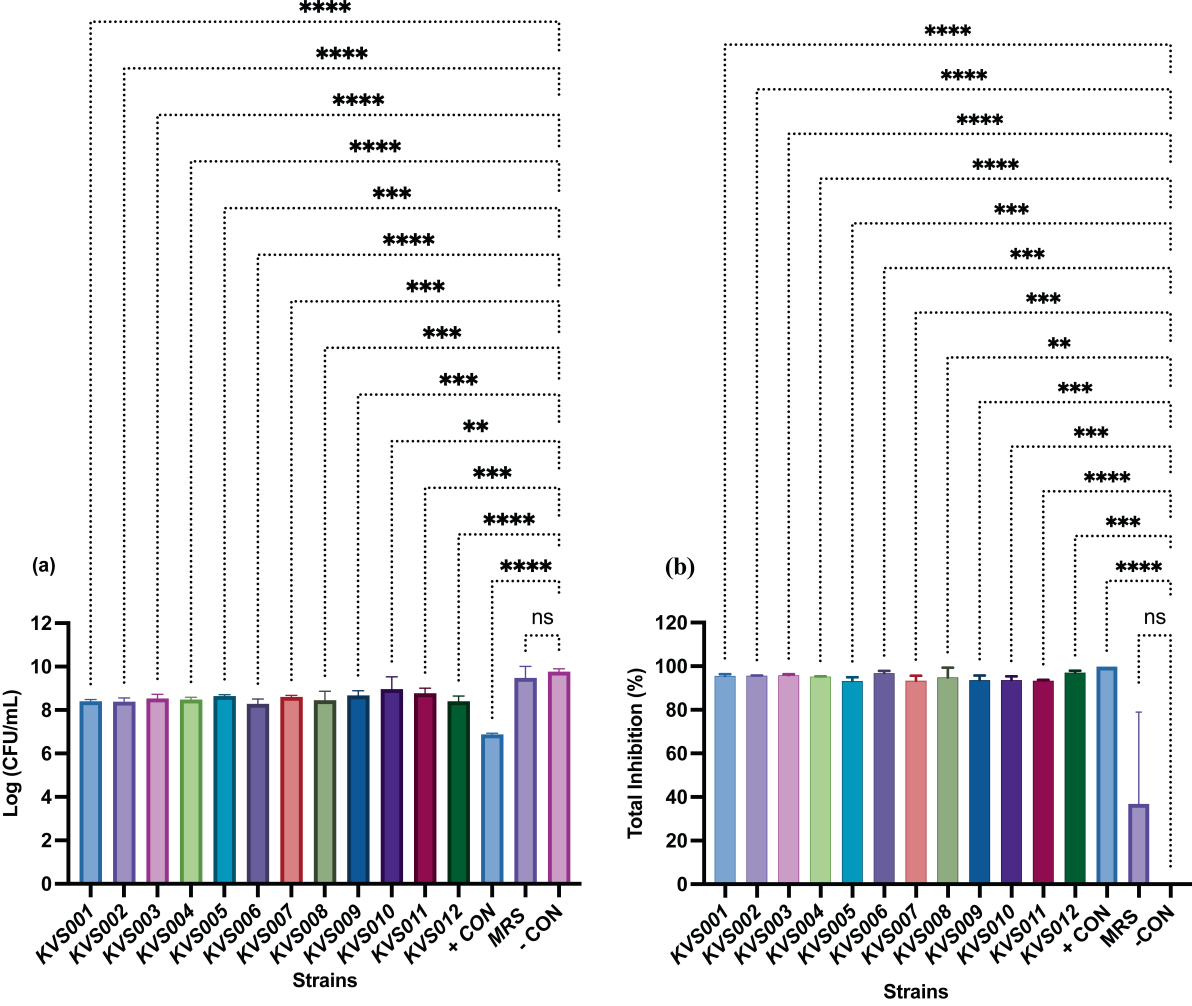
The antimicrobial activity of CFS produced by KVS strains against *G. vaginalis*. **(a)** Antimicrobial profile of KVS strains. **(b)** Total inhibition in comparison to negative control (- CON). *p*-values are represented as follows (**p ≤ 0.01, ***p ≤ 0.001, ****p ≤ 0.0001, and *ns* = not significant). Unless otherwise specified, significance was calculated relative to the designated control group. +CON (clindamycin 32 μg/mL), MRS (MRS + *G. vaginalis*), and - CON (PBS only).

Bacterial vaginosis is characterized by disruption of the normal vaginal microbiota and overgrowth of opportunistic pathogens, particularly *G. vaginalis*. We therefore, evaluated the antimicrobial activity of the KVS strains against this pathogen to assess their potential role in maintaining the vaginal microbial balance. The antimicrobial activity of the KVS strains against *G. vaginalis* is shown in Figure 5. The positive control (clindamycin, 32 μg/mL) inhibited the growth of *G. vaginalis* by 99.99% compared to the negative control (*p* < 0.05), the highest inhibition observed among all treatment groups. The inhibitory effect of the KVS strains on *G. vaginalis* was not significantly different from each other, however, the higher inhibition of 96.53, 95.81, and 95.80% against *G. vaginalis* was seen by KVS006, KVS002, and KVS001, respectively (*p* < 0.05). The strong inhibitory activity observed against *G. vaginalis* highlights the potential of selected KVS strains in promoting vaginal health.

#### Hydrogen peroxide measurement

**FIGURE 6:**
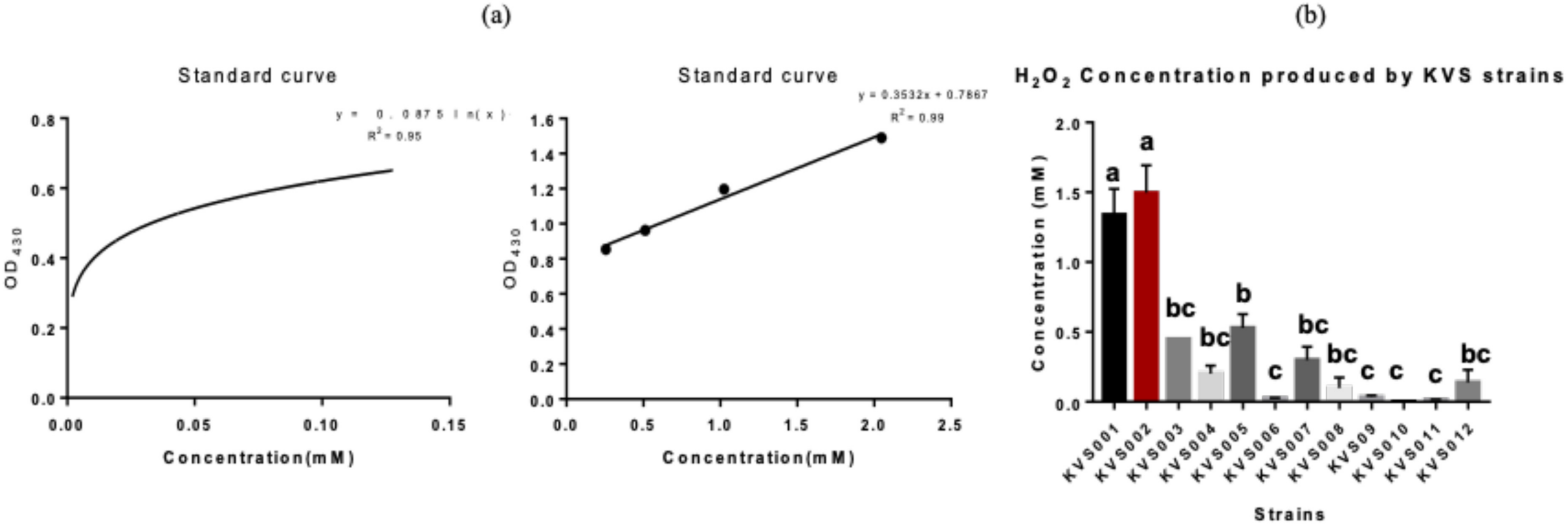
Quantification of hydrogen peroxide produced by KVS strains. **(a)** Standard calibration curve for hydrogen peroxide. **(b)** Concentration of hydrogen peroxide produced by KVS strains. Bars sharing a letter are not significantly different.

Hydrogen peroxide production is an important functional characteristic of vaginal probiotic bacteria, inhibits pathogenic bacterial growth and maintains the vaginal microbial balance. Here, we evaluated the hydrogen peroxide production of KVS strains as one of their indicators of probiotic potential as shown in Figure 6. Quantification was performed using absorbance at OD_430_, and interpolated from a standard calibration curve (Figure 6a). Substantial variability in hydrogen peroxide production was observed among the strains. Among all strains, KVS002 showed the highest hydrogen peroxide production, reaching 1.56 mM over 18 h of incubation. This level was significantly greater than that of other strains (*p* < 0.05). KVS001 also showed strong hydrogen peroxide production (1.40 mM/18 h), followed by KVS005 (approximately 0.52 mM/18 h) as shown in Figure 6b. The remaining strains produced intermediate concentrations, indicating strain dependent differences in production of hydrogen peroxide. The results suggest that potential of selected KVS strains in maintaining the vaginal microbial homeostasis. Thereby, inhibiting the growth of susceptible pathogenic microorganism.

#### Inhibition of the adhesion of *Gardnerella vaginalis* to the HeLa cell line

**FIGURE 7:**
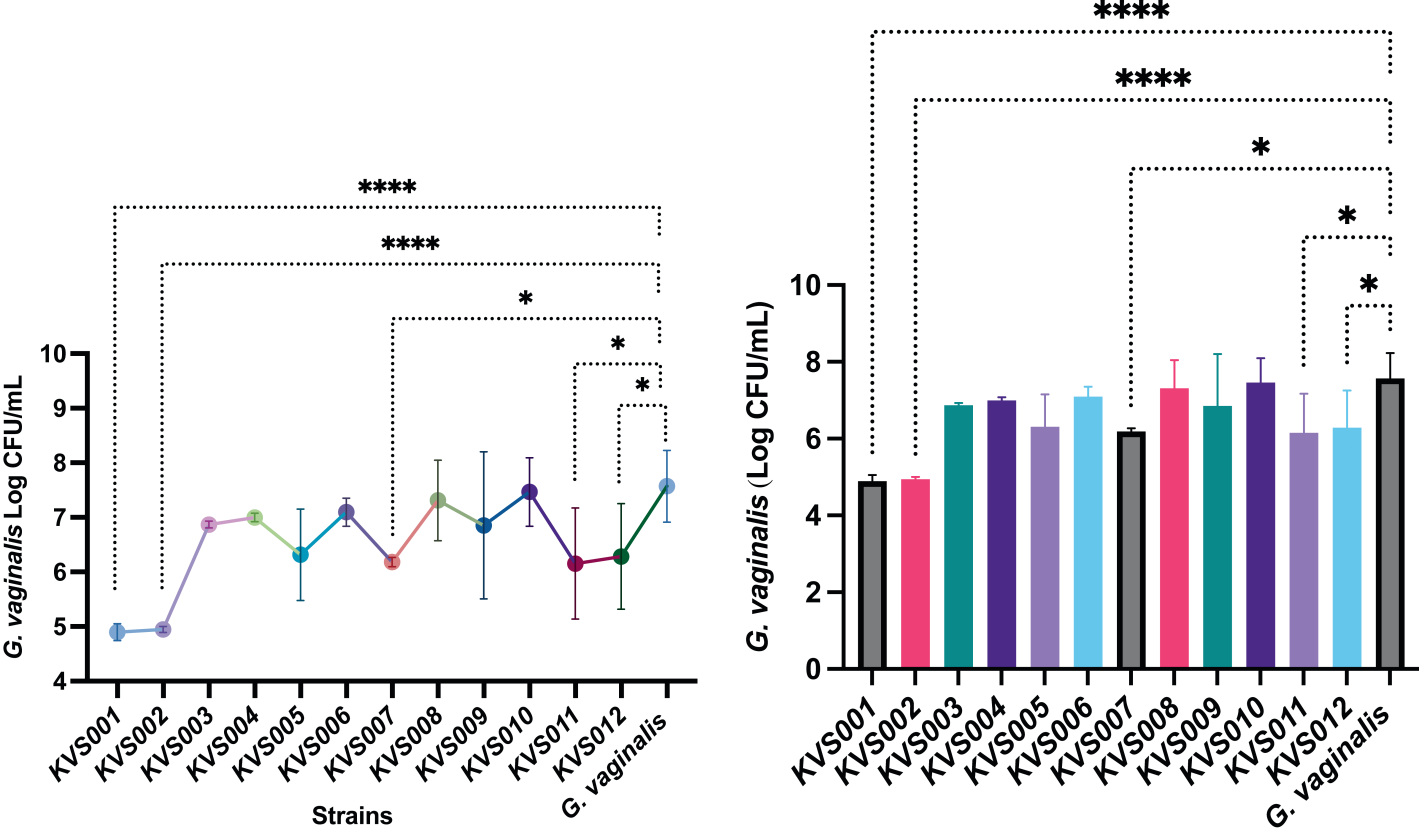
Inhibition of the adhesion of *G. vaginalis* on HeLa cells by KVS strains. Statistical significance was determined by one-way ANOVA followed my multiple comparison testing. *p*-values are indicated as (*p < 0.05, ****p < 0.0001). Strains not indicated show non-significant inhibition compared to control.

Bacterial vaginosis is mainly associated with adhesion and colonization of pathogenic microorganism to epithelial cells. Therefore, KVS strains were screened for their ability to interfere with pathogenic microbial adhesion. Figure 7 shows the result of the inhibition of the adhesion of *G. vaginalis* KCTC 5096 to HeLa cells by CFS of KVS strains. The CFS of KVS strains showed inhibitory activity against the adhesion on *G. vaginalis* to HeLa cells, with the most prominent effect observed for strain KVS001 and KVS002. These strains reduced *G. vaginalis* attachment by 1.6- and 1.65-fold as compared to the control (*p* < 0.05), suggesting a potential vaginal microbial balance by limiting the colonization of *G. vaginalis* on epithelial surfaces.

#### Adhesion to HeLa cell line

**FIGURE 8:**
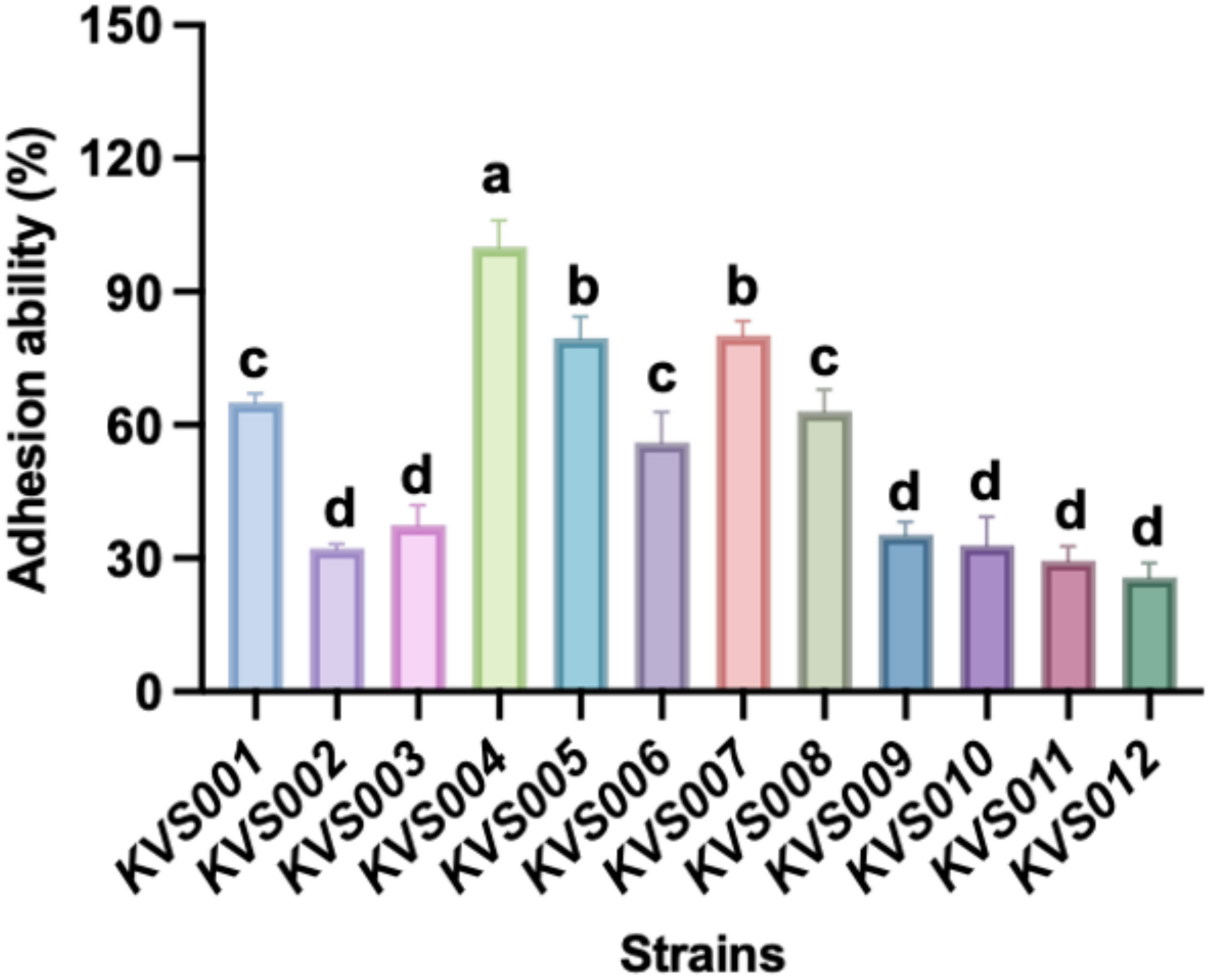
Adhesion ability of KVS strains to HeLa cells. Different letters (a–d) above bars indicate statistically significant differences among strains (one-way ANOVA, Tukey *post-hoc* test, *p* < 0.05). Bars sharing a letter are not significantly different.

Further, to assess the colonization of KVS strains, we evaluated their adhesion potential to HeLa cells as shown in Figure 8. The adhesion ability to HeLa cells was strain-dependent, and all strains attached to HeLa cells over or close to 50%. Strain KVS004 showed the highest adhesion ability, 107.33%, followed by KVS007 (102.00%) and KVS008 (89.05%) (*p* < 0.05).

## Discussion

In this study, 12 bacterial isolates were collected from the vagina of Korean women and designated as KVS (Korean female Vaginal Secretion) strains. These strains were then screened for efficacy in improving symptoms of PMS and BV through *in vitro* experiments. In addition, the efficacy of KVS strains were evaluated based on the guidelines set by the Korean Ministry of Food and Drug Administration, for recognition as health functional food for PMS and BV. As a result of assays, the following conclusions were drawn.

The present study was designed based on the reported association between microbiome dysbiosis and the development of PMS and BV. Researchers have found that PMS is related to high estrogen levels in the luteal phase, and these estrogen levels are associated with β-glucuronidase produced by the microbiota in the intestine (Ervin et al., 2019). In addition, the main cause of BV is due to the overgrowth of anaerobic bacteria and disruption of the normal vaginal microbiota (Chee et al., 2020). Therefore, it is expected that prevention and symptom improvement could be possible if dysbiosis is resolved. One of the most effective treatments of dysbiosis is through the administration of beneficial microbiota such as probiotics (Hashem and Gonzalez-Bulnes, 2022). The observed probiotic propoerties of the KVS strain support their potential role in modulating microbial communities associated with PMS and BV.

Before the evaluation of efficacies to improve and prevent PMS and BV *in vitro*, probiotic properties evaluations were performed on the KVS strains. The evaluations consisted of antibiotics susceptibility test, adhesion assay, and acid and bile tolerance test. Only 8 KVS strains passed the antibiotic susceptibility criteria suggested by EFSA; KVS001, KVS002, KVS004, KVS006, KVS008, KVS009, KVS010, and KVS011, therefore these strains are deemed safe to be used as commercialized probiotics.

Adhesion ability is one of the important characteristic of probiotic strains by facilitating colonization and persistence within mucosal environments. In most cases, auto-aggregation ability is related to cell adherence properties (Todorov et al., 2008; Tuo et al., 2013; Shen et al., 2023; Soleimani et al., 2023). In this study, all KVS strains exhibited auto-aggregation ability of more than 50%, suggesting a favorable colonization potential. In addition, KVS strains showed strong mucin adhesion ability, suggesting their adherence to mucus covered epithelial surfaces.

All KVS strains showed 1.85–30.65% of acid tolerance and 12.75–63.50% bile tolerance. Although acid tolerance is lower than bile tolerance, most KVS strains are expected to survive in the digestive tract.

Antimicrobial activity assay was performed to measure the growth inhibitory activity of KVS strains on pathogens causing PMS and BV; which include *E. coli*, *K. pneumoniae*, *A. baumannii*, *S. aureus*, *S. enterica*, *C. perfringens*, and *G. vaginalis*. These pathogens except *G. vaginalis* were found to produce β-glucuronidase which can activate estrogen and thereby escalating the severity of PMS. Also, the overgrowth of *G. vaginalis* is the main reason for the occurrence of BV. Therefore, inhibiting the growth of these pathogens would likely result in the reduction of symptoms and prevention of PMS and BV. The CFS of KVS strains significantly inhibited the growth of pathogens causing PMS and BV especially, strain KVS001 and KVS002 which showed consistent antimicrobial activity against all pathogens tested as compared to other KVS strains. Based on these results, strain KVS001 and KVS002 were expected to be the most effective microbiome modulator to inhibit the growth of pathogens causing PMS and BV.

Enzyme inhibition assay was performed to measure the inhibitory activity of KVS strains on beta-glucuronidase which is one of the causes of PMS. When a high amount of beta-glucuronidase is secreted by intestinal microbiota, the enzyme activates estrogen that is eventually re-uptake into the body, resulting in high estrogen levels that escalate the PMS severity. Therefore, inhibiting β-glucuronidase activity would be a possible mechanism for reducing the severity of PMS. As seen in this study, a total of 11 KVS strains showed inhibitory activity over 70% except for strain KVS006.

Hydrogen peroxide-producing capability is an important feature of lactic acid bacteria capable of maintaining a healthy vaginal microbiota (Pascual et al., 2006; Martinez et al., 2008; Sgibnev and Kremleva, 2015; Shen et al., 2023). Researchers found that *Lactobacillus* spp. may represent a nonspecific antimicrobial defense mechanism of the normal vaginal ecosystem (Osset et al., 2001; Pascual et al., 2006; Wasiela et al., 2009; Fuochi et al., 2019; Chee et al., 2020; Shen et al., 2023). In this study, strain KVS002 produced the highest amount of hydrogen peroxide, suggesting its potential to control the proliferation of BV associated pathogens such as *G. vaginalis*.

In addition, cell adhesion ability and inhibition of the adhesion of *G. vaginalis* to HeLa cells were evaluated using CFS produced by KVS strains. Vaginal lactic acid bacteria in healthy women can block the colonization of pathogenic microorganisms by occupying or blocking potential binding sites in the mucosa (Zárate and Nader-Macias, 2006). However, when the proportion of lactic acid bacteria in the vagina decreases, there is a tendency for the increment of BV causing anaerobic bacteria. Also, the adhesion of microorganisms to epithelial cells is an important step in forming and sustaining a cluster in a particular microenvironment such as the vaginal lining. Therefore, the lactic acid bacteria must confer cell adhesion ability as well as the ability to inhibit the adhesion of pathogens. Based on observations from this study, strain KVS001 and strain KVS002 significantly inhibited the adhesion of *G. vaginalis* to HeLa cells.

## Conclusion

The present study provides a rigorous functional assessment of probiotic isolates derived from the vaginal microbiota of healthy Korean women, with the aim of identifying strains capable of mitigating biological processes implicated in premenstrual syndrome. PMS has been mechanistically linked to elevations in estrogen recirculation, microbial dysbiosis, heightened β-glucuronidase activity, and the proliferation of anaerobic pathogens. The KVS strains investigated here exhibited a broad spectrum of probiotic properties that directly counter these pathological contributors. Multiple isolates demonstrated robust tolerance to acidic and bile conditions, supporting their capacity to survive gastrointestinal transit. Their high epithelial adhesion and strong auto-aggregation indicate substantial potential for mucosal colonization, a prerequisite for displacing pathogenic microorganisms and maintaining microbial equilibrium. Importantly, many KVS strains displayed significant antimicrobial activity against *G. vaginalis* and other PMS-associated pathogens, alongside potent inhibition of pathogen adhesion to epithelial cells. These findings highlight their ability to disrupt pathogen colonization and biofilm formation, both of which are central to dysbiosis-driven PMS exacerbation. The inhibition of β-glucuronidase activity observed in most isolates is particularly notable, as this enzyme facilitates estrogen deconjugation and intestinal reuptake, thereby contributing to elevated systemic estrogen levels. By suppressing this enzymatic activity, select KVS strains offer a plausible microbiome-mediated mechanism for reducing PMS severity. Additionally, the production of hydrogen peroxide by several strains further reinforces their antimicrobial and ecological competitiveness. Overall, among the KVS isolates evaluated, KVS001, and KVS002 exhibited superior probiotic characteristics, including strong antimicrobial activity against PMS and BV associated pathogens. In addition, these isolates showed strong inhibition of pathogen adhesion and favorable colonization related properties. However, KVS006 also showed promising activity, especially against *G. vaginalis*. All together, these results underpin KVS001 and KVS002 as strong candidates for development into probiotic formulations or functional foods aimed at restoring microbial balance and attenuating PMS-related symptoms. Future studies should focus on *in vivo* validation and clinical evaluation to determine their therapeutic efficacy, optimal dosage, and long-term safety profiles.

## Data Availability

The data presented in the study are deposited in the GENBANK repository, accession numbers (PZ546107, PZ546108, PZ546109, PZ546110, PZ546111, PZ546112, PZ546113, PZ546114, PZ546115, PZ546116, PZ546117, PZ546118).
